# High Performance of Biohydrogen Production in Packed-Filter Bioreactor via Optimizing Packed-Filter Position

**DOI:** 10.3390/ijerph18147462

**Published:** 2021-07-13

**Authors:** Chen-Yeon Chu, Jin-Long Zheng, Tsung-Hsien Chen, Prakash Bhuyar

**Affiliations:** 1 Program of Mechanical and Aeronautical Engineering, Feng Chia University, Taichung 407802, Taiwan; arlex.haogeng@gmail.com (T.-H.C.); 2 Master’s Program of Green Energy Science and Technology, Feng Chia University, Taichung 407802, Taiwan; ai52hebe@gmail.com; 3 Center for General Education, Feng Chia University, Taichung 407802, Taiwan; 4 School of Renewable Energy, Maejo University, Chiang Mai 50290, Thailand; mju6315301002@mju.ac.th

**Keywords:** hydraulic retention time (HRT), expired beverage drink, hydrogen production rate, hydrogen yield, *Clostridium* sp., high-strength organic effluent

## Abstract

In this present investigation, a packed-filter bioreactor was employed to produce hydrogen utilizing an expired soft drink as a substrate. The effects of feeding substrate concentrations ranging from 19.51, 10.19, 5.34, 3.48, to 2.51 g total sugar/L were examined, and the position of the packed filter installed in the bioreactor at dimensionless heights (h/H) of 1/4, 2/4, 3/4, and 4/4 was studied. The results revealed that with a substrate concentration of 20 g total sugar/L and a hydraulic retention time (HRT) of 1 h, a packed filter placed at the half-height position of the bioreactor (h/H 2/4) has the optimal hydrogen production rate, hydrogen yield, and average biomass concentration in the bioreactor, resulting in 55.70 ± 2.42 L/L/d, 0.90 ± 0.06 mol H_2_/mol hexose, and 17.86 ± 1.09 g VSS/L. When feeding substrate concentrations varied from 20, 10, to 5 g total sugar/L with the packed-filter position at h/H 2/4, *Clostridium* sp., *Clostridium tyrobutyricum*, and *Bifidobacterium crudilactis* were the predominant bacteria community. Finally, it was discovered that the packed-filter bioreactor can produce stable hydrogen in high-strength organic effluent.

## 1. Introduction

In recent years, considerable high-strength organic wastewater has been produced along with industrial beverage development. The annual beverage wastewater is abundant globally, and this organic wastewater pollutes rivers if improperly disposed of. Several investigators made a definition of high-strength organic wastewater as those of COD (chemical oxygen demand) concentration higher than 4000 mg/L [1,2,3]. The high-strength organic wastewaters treatment process still faces some obstacles for thoroughly treating the effluents to fit the requirement of constantly rising environmental standards [3]. In the early stage, the environmental researchers focused on how to treat the COD to fit the discharge standard [4,5,6]. Ren et al. [4] studied the optimized operational parameters in a submerged membrane bioreactor for high-strength organic wastewater treatment and found that the optimum operational parameters for the treatment of a high-strength traditional Chinese medicine wastewater were as follows: hydraulic retention time (HRT) was 5 h, SRT was 100 days, COD loading rate was less than 20.5 kg/m^3^/d, the range of Mixed Liquor Suspended Solids (MLSS) was 7543–13,694 mg/L. Sowmeyan and Swaminathan [5] used an inverse anaerobic fluidized bed reactor to treat high-strength organic wastewater and achieved 84% COD removal at an organic loading rate (OLR) of 35 kg COD/m^3^/d. Hamza et al. [6] treated high-strength organic wastewater to optimize the organics-to-nutrients (COD: N:P) ratio by aerobic granular sludge method and achieved overall COD removal efficiencies of 98%, at HRT of 8 h.

More post-treatment processes are required to meet wastewater discharge standards, which necessitates increased energy consumption. As a result, several researchers began to develop the anaerobic biohydrogen production (BHP) technology. The BHP technology treats high-strength organic wastewater and generates a clean energy carrier that is called biohydrogen [7,8]. Most of them are biohydrogen produced by the high organic pure substrate, which is higher than 15 g COD/L. Numerous researchers have successfully produced biohydrogen using a variety of organic wastes such as food wastes, molasses, cassava waste, algae waste, and water hyacinths [9,10,11,12,13]. Since the development of biohydrogen production, most of them are fed with 15–40 g COD/L (17.4–46.4 g total sugar/L) to produce hydrogen. However, many beverage processes have a concentration of about 2.5 to 10 g COD/L [14,15].

In the last ten years, these technologies have developed a trend toward the two-stage anaerobic digestion system, which can produce biohydrogen and biomethane simultaneously and achieve effective wastewater treatment [16,17,18]. Mari et al. [16] studied the energetic potential from cassava starch wastewater in a two-stage system (BioH_2_ + BioCH_4_) composed of anaerobic sequencing batch biofilm reactors (AnSBBR). They reported that the maximum methane productivity and yield of 2.71 L/L/d and 0.263 L/g COD were obtained at OLR of 12 g COD/L/d, and the estimated energy production rate was 105.2 kJ/L/d. Nguyen et al. [17] studied two-stage biohydrogen and biomethane productions in a continuous co-digestion process from a mixture of swine manure and pineapple waste and found the optimal total energy of 196.47 kJ/L/d and chemical oxygen demand (COD) removal efficiency of 90%. Kisielewska et al. [18] studied continuous biohydrogen and biomethane production from whey permeate in a two-stage fermentation process and found that the total chemical oxygen demand elimination from whey permeate reached 98% in a two-stage process.

This investigation aimed to examine the best substrate concentration and packed filter position to enhance the biohydrogen production. Different anaerobic digestion reactors lead to different yields and gas production performance under the same operating conditions. Therefore, finding the most suitable reactor and optimal operating conditions are also worthy of in-depth discussion. The packed-filter bioreactor was designed for testing the best position of the packed filter in the bioreactor. The feeding substrate concentration was also tested for checking the tolerance of high-strength organic wastewater level and biohydrogen production potential by using expired beverages.

## 2. Materials and Methods

### 2.1. Seed Sludge and Substrate

The anaerobic hydrogen-producing bacteria used in this study was obtained from the final sedimentation tank sludge of the community wastewater treatment plant in Li-Ming, Taichung City. The sludge was heat-treated at 100 °C for one hour to inhibit methane-producing bacteria and then implanted into the reactor for cultivation [19]. In this study, the high organic-based wastewater, and the processing wastewater from the beverage plant in Taoyuan, were used as carbon sources for hydrogen production. The wastewater samples were collected in the sampling glass bottles. Sampling was done at three different locations in the Taoyuan wastewater plant. The composition of the expired beverage wastewater was in total sugar of 110,000 mg/L, COD of 126,578 mg/L, and pH of 3.52, respectively.

### 2.2. Bioreactor Design

**Figure 1:**
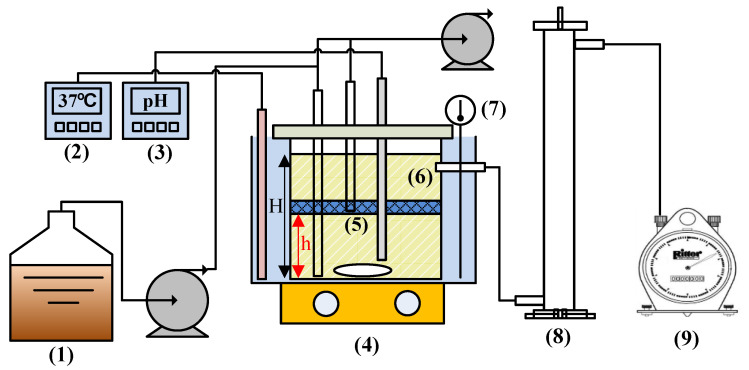
Schematic diagrams of an experimental process for M365-Blue bioreactor (M365-Blue). (1) Medium tank, (2) Temperature meter, (3) pH meter, (4) Magnetic stirrer, (5) Packed-Filter (h/H-1/2) (h: height from the bottom of the bioreactor; H: total height of working volume of the bioreactor), (6) H_2_ fermenter, (7) Thermometer, (8) Gas–liquid separator, (9) Wet gas meter.

The schematic diagrams of the experimental process for the M365-Blue bioreactor are represented in Figure 1. The M365-Blue Biohydrogen Production System was a continuous stirred anaerobic bioreactor (M365-Blue Bioreactor, Matala Water Technology Co., Ltd., Taichung City, Taiwan) 2:1 aspect ratio. The specification of commercial Flex (Matala Water Technology Co., Ltd., Taiwan) was an FSM365 type with a specific surface area of 365 ± 10 m^2^/m^3^, the thickness of 4 cm, the fiber diameter of 0.55 ± 0.1 mm and free volume of 94%, respectively. The bioreactor was made of transparent glass with a working volume of 2.5 L. The reactor was stirred with a magnet stone and contained a packed biofilter. The reactor was heated to a constant temperature by using a water bath and a heater. The temperature was controlled at 37 °C. The pH was monitored by using a pH controller (LP-3000; AI-ON Industry Co., Ltd., Taiwan) and 1M Na_2_CO_3_ as alkali at any time to monitor the appropriate addition and the reaction tank environment was maintained between pH 5.5~6.0.

### 2.3. Experimental Procedures

The reaction tank employed in this experiment was a 2.5-litre packed-filter (M365-Blue) biohydrogen reactor with high organic wastewater as the primary carbon source. A 2.5 L packed-filter (M365-Blue) bioreactor (PFBR) was started at an HRT of 8 h. After 35.5 h of acclimation, the pH of the reactor was reduced to 5.5. Then the HRT was adjusted to 1 h and substrate concentration of 19.51 ± 1.06 g total sugar/L (A Section). After that, the feeding substrate concentration was constantly reduced to 10.19 ± 1.68 (B section), 5.34 ± 1.05 (C section), 2.51 ± 0.76 (D section), and 3.48 ± 1.33 (E section) g total sugar/L. The biohydrogen reactor was used to investigate the effect of the packed-filter position on hydrogen concentration, hydrogen production rate, hydrogen yield, total sugar utilization and biomass concentrations at the different dimensionless heights of the bioreactor (h/H) 1/4, 2/4, 3/4, and 4/4, respectively. The full-index experiments were carried out with feeding substrate concentrations of 20.0, 10.0, 5.0, and 3.5 g total sugar/L and HRT 1 h.

The first experimental effect was tested on the substrate concentration in the packed-biofilter reactor. The feeding substrate concentration was varied from 20, 10, 5, 3.5, to 2.5 g total sugar/L. The packed biofilter was mounted at the middle height of the bioreactor that h/H equals 1/2 (h means height from the bottom of the bioreactor; H means the total height of working volume of the bioreactor). After that, the full-index experiments of packed-filter positions (h/H) were changed from 1/4, 2/4, 3/4, and 4/4 at different substrate concentrations of 20, 10, 5, and 3.5 g total sugar/L, respectively. The peristaltic pump was used to draw out the biomass sample for determining biomass concentrations in the bottom and filter. The effluent was used as a sample for calculating the biomass concentration in the bioreactor’s top layer

### 2.4. GC and DGGE Analysis

The gas composition was analyzed by Gas Chromatograph (SHIMADZU GC-14B) with TCD. The metabolites of lactic acid, formic acid, acetic acid, propionic acid, butyric acid and ethanol were analyzed by Liquid Chromatography (Shimadzu LC-10AT) with RID. Total sugar was determined using the phenol–sulfuric acid technique [20], with an absorbance wavelength of 485 nm in a UV/VIS spectrophotometer (Jasco V-530). The biomass weight was measured by the APHA method. Bacterial community structure was examined by denaturing gel gradient electrophoresis (DGGE) targeting 16S rRNA genes.

## 3. Results

### 3.1. Effect of Substrate Concentration

**Figure 2:**
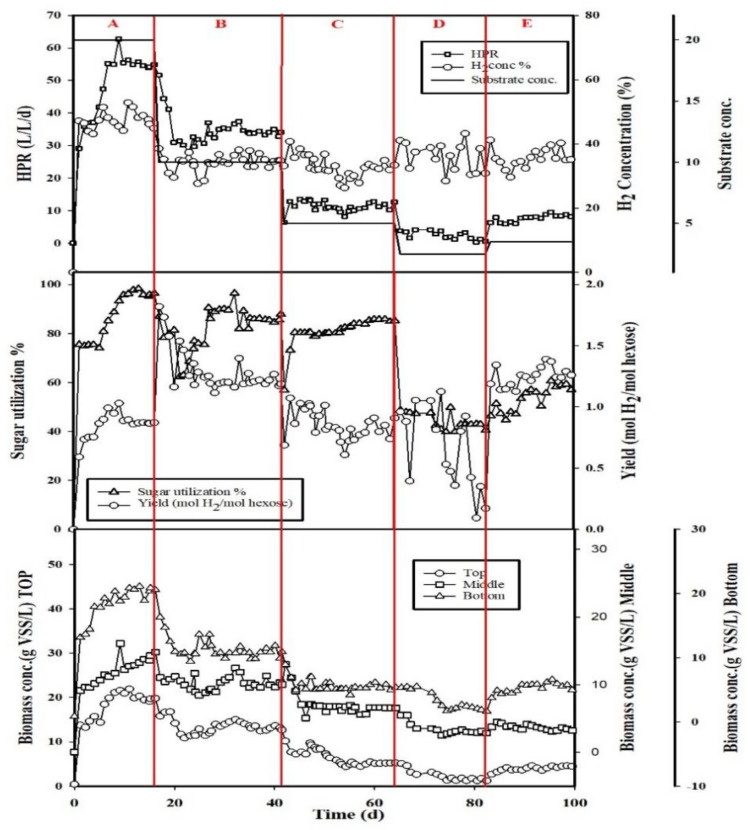
The performance of the hydrogen production evolution under different substrate concentrations with HRT 1 h and h/H 0.5.

Figure 2 demonstrates the performance of the hydrogen production evolution under different substrate concentrations with HRT 1 h and h/H 0.5. When the feeding substrate concentration was reduced to 2.51 ± 0.76 g total sugar/L, the hydrogen production yields up and down dramatically, and total sugar utilization was only 0.46 ± 0.27 mol H_2_/mol hexose in section D. It means that the system cannot run well in the condition of feeding substrate concentration lower than 2.5 g total sugar/L [18]. The feeding substrate was then adjusted to 3.5 g total sugar/L (E section), and the steady state was reached after 99 days; the hydrogen concentration and the HPR returned to the average values of 35.02 ± 3.73% and 8.35 ± 0.55 L/L/d, respectively. There was no significant difference in the composition of the liquid metabolites, but with the increase of the substrate concentration, the yield of ethanol decreased. It means that the system favors producing the biohydrogen in the high feeding substrate concentration over the low substrate concentration, which was similar to the literature reports from Chu et al. [21] and Tran et al. [22]: the biohydrogen production increased with the increase in feeding substrate concentration. A lower feeding substrate concentration resulted in slower cell growth, which can be attributed to insufficient carbon source availability. The bacterial growth increased with an increase in substrate concentration up to 60 g total sugar/L, and after that, the substrate inhibition effect appeared for any further increase in substrate concentration in sugar-rich substrate. However, the increase of concentration COD up to 50 g COD/L was inefficient on productiveness by lignocellulose materials.

The steady-state results of hydrogen concentration, hydrogen production rate (HPR), biohydrogen production yield, the total sugar utilization, and the biomass concentrations in the top, middle, and bottom of the reactor are shown in Table 1. As shown in Figure 2 and Table 1 that after 16 days of operation, the hydrogen concentration and HPR reached peak values of 48.00 ± 3.03% and 54.98 ± 0.81 L/L/d, respectively. The yield of the biohydrogen production was 0.90 ± 0.06 mol H_2_/mol hexose, and the sugar utilization was 96.43 ± 1.05%. The results showed that the biomass concentrations were 20.14 ± 0.91, 13.45 ± 0.85, and 20.33 ± 0.78 g VSS/L in the top, middle, and bottom of the reactor. The hydrogen concentrations ranged from 31.86 ± 2.17% to 35.02 ± 3.73% in sections B to E. The hydrogen production rates decreased from 34.42 ± 1.27 to 1.60 ± 0.96 L/L/d with the similar trend of hydrogen production yield from 1.21 ± 0.03 to 0.46 ± 0.27 mol H_2_/mol hexose when feeding substrate concentration decreased from 10.19 ± 1.68 to 2.51 ± 0.76 g total sugar/L. The phenomenon mentioned above also obviously found that the values of biomass concentrations trend were the same as the feeding substrate concentration in the top, middle, and bottom of the reactor.

Table 2 shows the composition of liquid metabolites in the different substrate concentrations with the HRT 1 h and the packed filter in the middle height of the bioreactor. It was shown that the butyric acid and acetic acid accounted for about 86.99–75.39% of total soluble microbial products (SMP) from the substrate concentration varied from 19.51 ± 1.06 to 2.51 ± 0.76 g total sugar/L. It looks like a typical butyric acid production pathway took place in the system. When butyric acid is produced in the fermentation, it is obvious that the production of by-product acetic acid is a branched pathway, which complicates the recovery of butyrate in downstream processing [23]. If the butyrate production pathway is constrained by excess butyrate, and H_2_ formation is unfavorable due to large amounts of dissolved hydrogen, no H_2_ is produced. In contrast, if the acetate production pathway is obstructed by abundant acetate or high dissolved hydrogen concentrations, the bacteria can still renew NAD^+^ via butyrate production [24].

### 3.2. Effect of Packed-Filter Position

**Figure 3:**
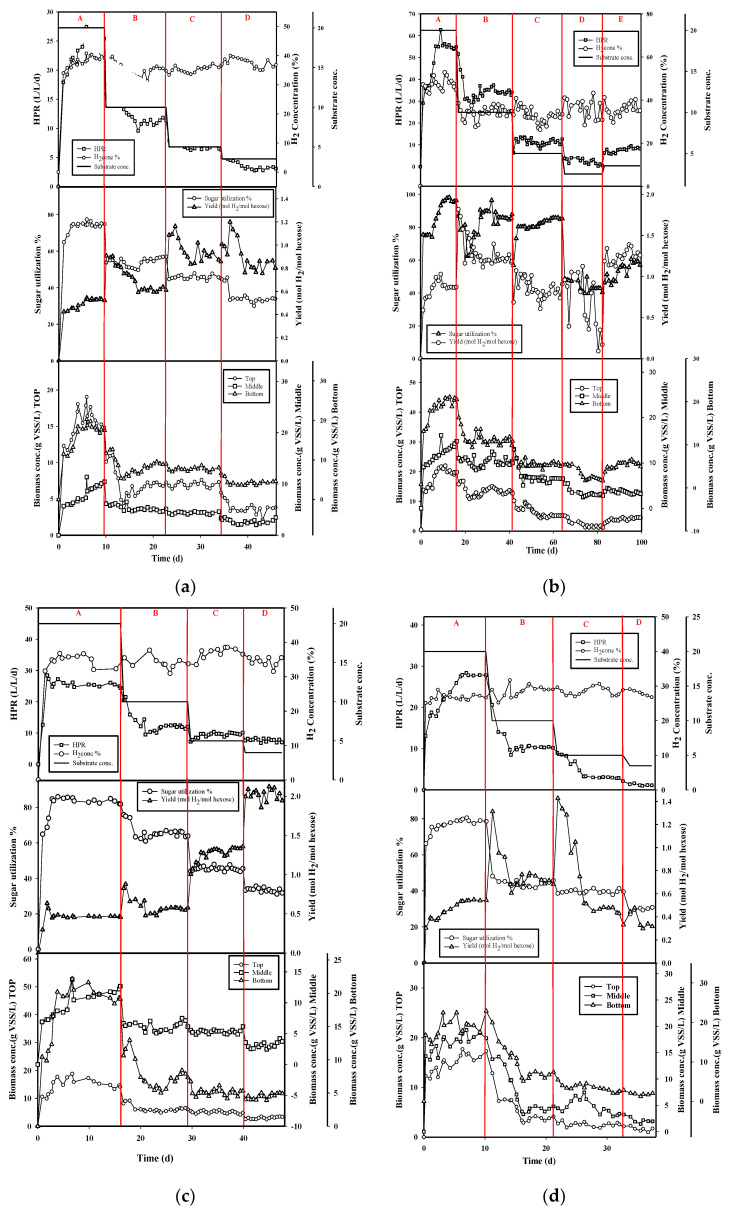
The hydrogen production and biomass concentration evolutions in the four positions from the bottom of the bioreactor: (**a**) h/H 1/4, (**b**) h/H 2/4, (**c**) h/H 3/4, (**d**) h/H 4/4.

As can be seen from Figure 3a–d, the packed-filter biohydrogen bioreactor is beneficial to the growth of the bacteria and does not lead to bacteria washout due to the system being able to run smoothly at a relatively low hydraulic retention time, such as HRT 1 h. Employing a packed filter in the bioreactor for the purpose of increasing biohydrogen production may be a more straightforward and cost-effective operation strategy in the long run [25]. The appropriate block stays in the hydrogen-producing trough so that the hydrogen production rate has increased significantly. Figure 3a–d and Table 3 show the hydrogen production performance and biomass concentration in the four positions from the bottom of the bioreactor. Figure 3a–d and Table 3 show that the hydrogen production rate and hydrogen yield are significantly increased by increasing feeding substrate concentration on the different positions of the packed filter in the bioreactor. As shown in Table 3, the peak hydrogen production (HPR) of 55.70 ± 2.42 L/L/d was obtained at h/H 2/4 packed-filter position in the bioreactor with average hydrogen concentration 47.67 ± 2.66%, hydrogen yield 0.90 ± 0.06 mol H_2_/mol hexose when feeding substrate concentration of 20 g total sugar/L, and total sugar utilization of 94.42 ± 3.99%, respectively. The biomass concentration distribution at the top, filter, and bottom were 20.34 ± 0.92, 13.32 ± 1.41, and 20.01 ± 0.96 g VSS/L, respectively, with the average biomass concentration of 17.86 ± 1.09 g VSS/L, while the feeding substrate concentration was 20 g total sugar/L and the packed filter was placed at the half-height position of the bioreactor (h/H 2/4). As shown in Table 3, along with the packed-filter positions (h/H) that were varied from 1/4 to 4/4 at 20 g total sugar/L, the biomass concentration was ranged from 15.68 to 20.34 g VSS/L and 18.22 to 20.01 g VSS/L at the top and bottom of the bioreactor, respectively. A similar trend also occurred in other feeding substrate concentrations, which ranged from 3.5 to 10 g total sugar/L. However, a dramatic increasing of the biomass concentration occurred when the feeding substrate concentration varied from 10 to 20 g total sugar/L. It seems that the aggregated bacteria in the packed filter bioreactor grew up very well at a high organic loading rate (HRT 1 h). The washed-out phenomena did not appear in this high-speed feeding rate when packed filter was mounted in the bioreactor. As shown in Figure 3a–d, in the whole comparison, the peak hydrogen production efficiency at the position of the backed filter at h/H 2/4 was obtained since a higher average biomass concentration occurred at the h/H 2/4 position in the same feeding substrate concentration in different packed-filter positions.

The composition of the metabolites in the biohydrogen reactor under different positions of the packed filter is shown in Table 4. The ratio of butyric acid and acetic acid to the total SMP ranged from 85.60 to 70.31% for the four packed-filter positions at different substrate concentrations. The composition of the liquid metabolites showed that the system was mainly metabolized by the hydrogen-producing pathway of butyric acid and the typical butyric acid-producing pathway. According to theoretical calculations, for metabolic pathways to acetic acid, 1 mole of glucose by biotransformation can be converted entirely to 4 moles of hydrogen, and for metabolic path to the butyric acid pathway, 1 mole of glucose by biological conversion can be completely converted to 2 moles of hydrogen. Fang and Liu [21], Morimoto et al. [22], and Ueno [26] suggested that the highest hydrogen yield using glucose conversion is about 2.0–2.4 mol H_2_/mol glucose [26,27,28], and the result of low yields may be that the microorganisms use some of the metabolic pathways of glucose degradation to butyrate instead of acetic acid [26]. Lin et al. [29] showed that even if the glucose degradation rate was above 95%, the yield could be lower than 1.7 mol H_2_/mol glucose, because it is not only metabolized to the acetate or butyrate pathway, but also byproducts such as propionic acid, lactic acid, and ethanol. It can be seen that microbial metabolic pathways are the main factors causing the yield to be lower than the theoretical value. In the present study, the volatile fatty acids of the liquid metabolites produced in the hydrogen-producing bacteria are shown in Table 4. The TVFA was mainly composed of butyric acid and acetic acid, and the non-volatile fatty acid was separated by ethanol. In addition, the composition of the liquid metabolites showed that this system was the primary metabolic pathway of butyric acid for hydrogen production. Lactic acid-producing microbes need pyruvic acid as a precursor to produce lactic acid. Table 4 demonstrates the sufficient availability of lactic acid around 3%. Currently, lactic acid is regarded as the most promising feedstock monomer for chemical conversions. Lactic acid can undergo several chemical reactions to create potentially useful compounds since it has two reactive functional groups, a carboxylic group and a hydroxyl group. The advancement and expansion of the lactic acid production biotechnology technologies for the synthesis of bio-based lactic acid derivatives may eventually replace chemically generated methods [30,31].

### 3.3. Microbial Community Analysis

**Figure 4:**
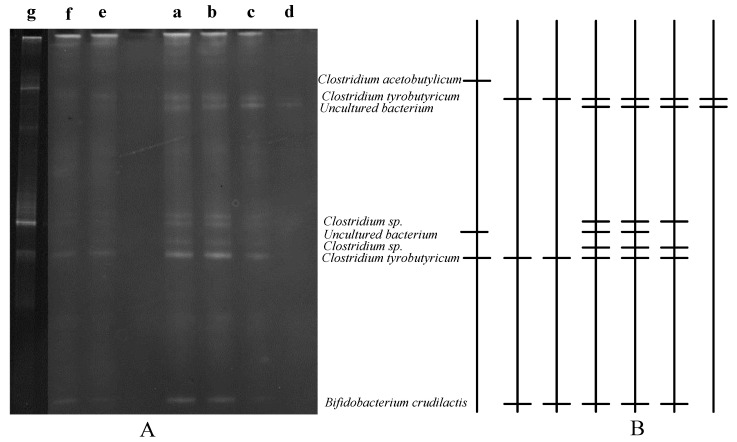
Microbial community diversity in packed-filter hydrogen production bioreactor under different operating conditions. (**A**) DGGE banding of PCR-amplified 16S rRNA gene from microbial DNA isolated, (**B**) patterns diagram, where **a**–**d** represent the feeding substrate concentration of 20, 10, 5, and 3.5 total sugar g/L when the position of the bioreactor was at h/H 2/4, whereas **e**–**g** represent the packed-filter position in the bioreactor (h/H) at 3/4, 1/4, and 4/4 when the feeding substrate concentration was 20 total sugar g/L.

Figure 4 shows the microbial community diversity of hydrogen production packed-filter bioreactor under different operating conditions. The dominant bacteria in this study are *Clostridium tyrobutyricum*, *Clostridium* sp., and *Clostridium acetobutylicum*. The obligate anaerobic bacterium *Clostridium* is one of the common hydrogen-producing bacteria suitable for living in a mesophilic environment that can use various carbohydrates to produce hydrogen. *Clostridium butyricum*, *Clostridium thermobutyricum*, *Clostridium tyrobutyricum*, *Clostridium acetobutylicum*, and *Clostridium pasteurianum* are the most common strains of *Clostridium* species, and most of them are mainly favorable to produce acetic acid and butyric acid [32,33,34,35].

As displayed in Figure 4, *Clostridium* sp., *Clostridium tyrobutyricum,* and *Bifidobacterium crudilactis* were the leading bacteria community when feeding substrate concentration varied from 20 to 3.5 g total sugar/L with the packed-filter location at h/H 2/4. The hydrogen production rate decreased when the substrate concentration decreased to 3.5 g total sugar/L, because the bright band of *Clostridium* sp. and *Bifidobacterium crudilactis* disappeared, as shown by DGGE analysis in Figure 4. Only *Clostridium tyrobutyricum* remained in the bioreactor, and this was since most of the other hydrogen-producing bacteria washed out, thereby affecting the performance of hydrogen production.

Figure 4 illustrates when the packed filter was at the position (h/H) at 1/4 in the bioreactor and 3/4 with the feeding substrate concentration of 20 g total sugar/L. The bacterial community were mainly *Clostridium tyrobutyricum* and *Bifidobacterium crudilactis*. The hydrogen-producing *Clostridium* sp. disappeared. From Table 3, it was found that the biomass concentration of these two conditions decreased a lot, resulting in inadequate hydrogen production. In the h/H 4/4 position, *Clostridium tyrobutyricum* was dominant, and the bright band of *Clostridium acetobutylicum* appeared. As shown in Table 3, the average biomass concentration was similar at the **a** (h/H 4/4) and **g** (h/H 2/4) operating conditions, which were 17.91 and 17.89 g VSS/L, respectively. However, the hydrogen production rate was 2 times larger when the packed-filter position was at h/H 2/4 than that at h/H 4/4. It can be presumed that the main bacteria in the position of the packed filter at h/H 4/4 may be *Clostridium acetobutylicum*. Some references point out that *Clostridium acetobutylicum* can provide covert glucose to produce acetic acid and butyric acid and produce acetone, butanol, and ethanol [36,37]. As shown in Table 4, ethanol is more abundant in the packed filter h/H 4/4 than the others. Due to different metabolic pathways of bacteria, resulting in **g** condition hydrogen yield and hydrogen production rate was lower than **a** condition, even the average biomass concentration was similar. The new reactor geometries should also be intended to increase SRT and mass transfer properties, allowing researchers to better understand the microbe–microbe contact interaction [38,39].

## 4. Conclusions

Fermentative synthesis of chemicals and biopolymers from waste and byproduct streams is an important research area with promising industrial applications. The effects of substrate concentration and varied packed-filter positions in a bioreactor on hydrogen generation performance were explored in this study. The butyric acid and acetic acid reported for about 86.99–75.39% of total soluble microbial products (SMP) from the substrate concentration varied from 19.51 ± 1.06 to 2.51 ± 0.76 g total sugar/L. The system prefers to produce biohydrogen in high feeding substrate concentrations over low feeding substrate concentrations, and the composition of the liquid metabolites revealed that this system was the primary butyric acid metabolic pathway for hydrogen generation. The peak of hydrogen production (HPR) of 55.70 ± 2.42 L/L/d was obtained at h/H 2/4 packed-filter position in the bioreactor with average hydrogen concentration 47.67 ± 2.66%, hydrogen yield 0.90 ± 0.06 mol H_2_/mol hexose with feeding substrate concentration of 20 g total sugar/L and total sugar utilization of 94.42 ± 3.99%, respectively. At the same time, the biomass concentrations at the top, middle and bottom positions of the bioreactor were 20.34 ± 0.92, 13.32 ± 1.41, and 20.01 ± 0.96 g VSS/L, respectively. The *Clostridium* sp., *Clostridium tyrobutyricum*, and *Bifidobacterium crudilactis* were the leading bacteria community in the packed filter bioreactor. Lastly, optimizing the packed-filter position and substrate concentration can improve hydrogen production; the greater the specific surface area, the higher the hydrogen production rate. However, the system must still be assessed and improved to ensure commercial viability.

## Figures and Tables

**Table 1:** Hydrogen production performance at different substrate concentrations (HRT 1 h; h/H 0.5).

Substrate Conc. (g Total Sugar/L)	Hydrogen Conc. (%)	HPR (L/L/d)	Yield (mol H_2_/mol hexose)	Total Sugar Utilization (%)	Biomass Conc. Top (g VSS/L)	Biomass Conc. Filter (g VSS/L)	Biomass Conc. Bottom (g VSS/L)
19.51 ± 1.06	48.00 ± 3.03	54.98 ± 0.81	0.90 ± 0.06	96.43 ± 1.05	20.14 ± 0.91	13.45 ± 0.85	20.33 ± 0.78
10.19 ± 1.68	34.77 ± 1.69	34.42 ± 1.27	1.21 ± 0.03	85.87 ± 1.91	13.52 ± 0.76	10.33 ± 0.91	10.77 ± 0.68
5.34 ± 1.05	31.86 ± 2.17	11.03 ± 1.22	0.82 ± 0.07	84.81 ± 0.99	5.83 ± 1.32	6.53 ± 0.40	5.32 ± 0.45
2.51 ± 0.76	34.40 ± 5.03	1.60 ± 0.96	0.46 ± 0.27	42.72 ± 3.02	1.49 ± 0.27	3.00 ± 0.18	2.08 ± 0.34
3.48 ± 1.33	35.02 ± 3.73	8.35 ± 0.55	1.31 ± 0.07	56.57 ± 2.92	4.27 ± 0.33	3.53 ± 0.35	5.76 ± 0.76

**Table 2:** Composition of liquid metabolites for hydrogen production in different substrate concentrations (HRT 1 h; h/H 0.5).

Substrate Conc. (g Total Sugar/L)	HLa (%)	HFo (%)	HAc (%)	HPr (%)	EtOH (%)	HBu (%)	(HAc + HBu) /SMP (%)	TVFA (mg COD/L)	SMP (mg COD/L)	TVFA/SMP
19.51 ± 1.06	1.09	0.11	12.97	1.43	10.38	74.02	86.99	8707.11	9835.21	88.53
10.19 ± 1.68	2.13	0.09	9.71	0.84	11.34	75.89	85.60	4903.98	5667.38	86.53
5.34 ± 1.05	1.14	0.38	9.77	6.09	15.89	66.73	76.50	2981.72	3593.73	82.97
2.51 ± 0.76	1.01	0.33	9.66	6.05	17.22	65.73	75.39	797.18	974.91	81.77
3.48 ± 1.33	1.49	0.56	7.31	1.2	19.97	69.47	76.78	1062.49	1352.81	78.54

HLa: lactic acid; HFo: formic acid; HAc: acetic acid; HPr: propionic acid; HBu: butyric acid; EtOH: ethanol; TVFA (total volatile fatty acid) = HFo + HAc + HPr + HBr; SM P(soluble microbial products) = TVFA + HLa + EtOH.

**Table 3:** Hydrogen production performance and biomass concentrations in the bioreactor at different packed-filter positions and feeding substrate concentrations with HRT 1 h.

Height (h/H)	Substrate Conc. (g Total Sugar/L)	Hydrogen Conc. (%)	Production Rate (L/L/d)	Hydrogen Yield (mol H_2_/mol hexose)	Total Sugar Utilization (%)	Biomass Conc. (g VSS/L) Top	Biomass Conc. (g VSS/L) Filter	Biomass Conc. (g VSS/L) Bottom
1/4	20.0	39.22 ± 1.14	25.76 ± 0.81	0.53 ± 0.01	74.83 ± 1.15	16.21 ± 1.48	9.23 ± 1.28	18.22 ± 1.01
	10.0	37.55 ± 0.79	11.11 ± 0.64	0.61 ± 0.01	43.70 ± 1.53	6.93 ± 0.62	5.00 ± 0.24	8.28 ± 1.02
	5.0	34.55 ± 0.89	6.78 ± 0.43	0.90 ± 0.05	39.76 ± 1.16	6.70 ± 0.49	4.29 ± 0.34	7.59 ± 0.55
	3.5	32.18 ± 0.98	3.10 ± 0.24	0.81 ± 0.04	29.56 ± 1.31	3.48 ± 0.57	2.59 ± 0.40	4.16 ± 0.39
2/4	20.0	47.67 ± 2.66	55.70 ± 2.42	0.90 ± 0.06	94.42 ± 3.99	20.34 ± 0.92	13.32 ± 1.41	20.01 ± 0.96
	10.0	34.77 ± 1.69	34.42 ± 1.27	1.21 ± 0.03	85.87 ± 1.91	13.52 ± 0.76	10.33 ± 0.91	10.77 ± 0.68
	5.0	31.86 ± 2.17	11.03 ± 1.22	0.82 ± 0.07	84.81 ± 0.99	5.27 ± 0.61	6.50 ± 0.40	5.76 ± 0.46
	3.5	35.02 ± 3.73	8.35 ± 0.55	1.31 ± 0.07	56.57 ± 2.92	4.21 ± 0.37	3.5 ± 0.33	5.32 ± 0.44
3/4	20.0	34.59 ± 1.80	25.34 ± 0.60	0.46 ± 0.01	83.72 ± 1.35	15.68 ± 1.63	11.20 ± 1.55	19.96 ± 1.10
	10.0	30.94 ± 5.25	11.67 ± 0.81	0.56 ± 0.01	65.33 ± 1.24	5.83 ± 0.53	6.19 ± 0.75	6.89 ± 1.12
	5.0	45.08 ± 1.49	9.69 ± 0.40	1.31 ± 0.03	45.59 ± 1.15	5.06 ± 0.47	5.35 ± 0.31	5.09 ± 0.48
	3.5	49.14 ± 2.00	7.59 ± 0.51	2.03 ± 0.08	33.00 ± 1.24	3.13 ± 0.33	3.24 ± 0.51	4.68 ± 0.37
4/4	20.0	26.64 ± 0.62	27.87 ± 0.26	0.54 ± 001	79.00 ± 0.98	16.59 ± 0.75	18.06 ± 0.84	19.09 ± 1.91
	10.0	28.98 ± 1.40	10.26 ± 0.37	0.72 ± 0.04	54.29 ± 2.65	3.96 ± 0.94	4.61 ± 1.09	7.15 ± 1.71
	5.0	28.78 ± 1.31	2.96 ± 0.33	0.46 ± 0.05	45.75 ± 1.16	2.41 ± 0.38	4.27 ± 1.50	3.06 ± 0.73
	3.5	27.94 ± 0.86	1.24 ± 0.22	0.37 ± 0.07	33.28 ± 1.50	1.56 ± 0.44	2.05 ± 0.45	1.79 ± 0.30

**Table 4:** Composition of the metabolites in the biohydrogen reactor under different packed-filter positions.

h/H	Substrate Conc. (g Total Sugar/L)	HLa (%)	HFo (%)	HAc (%)	HPr (%)	EtOH (%)	HBu (%)	(HAc + HBu) /SMP (%)	TVFA (mg COD/L)	SMP (mg COD/L)	TVFA/SMP
1/4	10.0	3.30	1.54	9.43	1.57	19.78	64.38	73.81	2404.95	3126.56	76.92
	5.0	1.42	1.27	7.46	2.61	23.53	63.71	71.17	1006.60	1341.24	75.05
	3.5	4.51	0.26	8.57	1.37	21.82	63.47	72.04	612.47	831.37	73.67
2/4	10.0	2.13	0.09	9.71	0.84	11.34	75.89	81.31	4903.98	5667.38	82.20
	5.0	1.14	0.38	9.77	6.09	15.89	66.73	66.42	2981.72	3593.73	72.04
	3.5	1.49	0.56	7.31	1.2	19.97	69.47	69.98	1062.50	1352.81	71.58
3/4	10.0	2.41	2.16	6.37	2.49	18.76	67.81	74.18	3130.21	3970.83	78.83
	5.0	4.62	0.92	6.45	2.79	17.09	68.13	74.58	1496.45	1911.42	78.29
	3.5	1.03	1.56	6.72	2.99	23.76	63.94	70.66	702.09	933.51	75.21
4/4	10.0	1.93	2.15	7.37	4.92	20.69	62.94	70.31	2523.52	3261.21	77.38
	5.0	0.39	1.21	7.83	3.79	23.67	63.11	70.94	1470.37	1936.23	75.94
	3.5	2.66	1.18	8.34	1.49	22.58	63.75	72.09	765.36	1023.75	74.76

HLa: lactic acid; HFo: formic acid; HAc: acetic acid; HPr: propionic acid; HBu: butyric acid; EtOH: ethanol; TVFA (total volatile fatty acid) = HFo + HAc + HPr + HBr; SM P(soluble microbial products) = TVFA + HLa + EtOH.

## Data Availability

Not applicable.
